# Tumeur stromale gastrointestinale de l'iléon avec rechute métastatique développée dans le mésentère

**DOI:** 10.11604/pamj.2014.18.18.4119

**Published:** 2014-05-05

**Authors:** Fanomezantsoa Raherinantenaina, Fanjandrainy Rasoaherinomenjanahary, Andriamampionona Tsitohery Francine, Andrianisa Hoby Rambel, Harinirina Yoël Honora Rantomalala, Luc Hervé Samison, Hery Nirina Rakoto Ratsimba

**Affiliations:** 1 Service de Chirurgie Générale et Vasculaire, CHUJRA (Centre Hospitalo-universitaire Joseph Ravoahangy Andrianavalona), Antananarivo, Madagascar; 2 Service de Chirurgie Viscérale B, CHUJRA, Antananarivo, Madagascar; 3 Laboratoire d'Anatomopathologie, CHUJRA, Antananarivo, Madagascar; 4 Service d'Urologie, CHUJRA, Antananarivo, Madagascar

**Keywords:** Imatinib, iléon, mésentère, tumeur stromale gastrointestinale, Imatinib, ileum, mesentery, gastrointestinal stromal tumor

## Abstract

Nous rapportons le cas d'un homme de 29 ans admis pour une masse hypogastrique très douloureuse. La laparotomie exploratrice réalisée en urgence permettait de mettre en évidence l'origine et la localisation iléale de la masse tumorale. Le traitement chirurgical consistait en une tumorectomie complète avec respect des marges carcinologiques. Les suites opératoires étaient simples et l'examen histologique confirmait la nature stromale de la tumeur iléale réséquée. Les marges de résection passaient en tissus sains. Le patient était perdu de vue et n'ayant reçu aucun traitement adjuvant. Un an plus tard, il est revenu pour ballonnement et masse pelviens d’évolution rapidement progressive et dont l'exploration chirurgicale révélait l'existence d'une tumeur mésentérique. Une tumorectomie était réalisée mais incomplète à cause d'une perte sanguine avec instabilité hémodynamique induite par l'exérèse tumorale. Après analyse histologique et immunohistochimique de la pièce opératoire, le diagnostic d'une tumeur stromale était confirmé. Il s'agissait d'une rechute métastatique à localisation mésentérique d'une tumeur stromale digestive d'origine iléale à fort potentiel malin. La réduction tumorale suivie d'un complément thérapeutique par l'Imatinib (glivec^®^) permettaient d'obtenir un résultat satisfaisant. Avec un recul de 12 mois, le patient était asymptomatique et aucune récidive locale ni de métastase à distance n'a été observée.

## Introduction

Les tumeurs stromales gastrointestinales ou GIST sont des pathologies relativement fréquentes bien connues en termes de chirurgie digestive et de thérapeutique ciblée [1]. Actuellement, leur prise en charge est bien codifiée avec la disponibilité des moyens diagnostiques et thérapeutiques sophistiqués facilement accessibles surtout dans les pays développés [2, 3]. L'objectif de ce travail était de rapporter le cas d'une pathologie chirurgicale prise en charge dans un pays à faible ressources. Il s'agissait d'une GIST iléale diagnostiquée sur le plan immunohistochimique de façon rétrospective à partir d'une rechute métastatique développée dans le mésentère.

## Patient et observation

**Figure 1:**
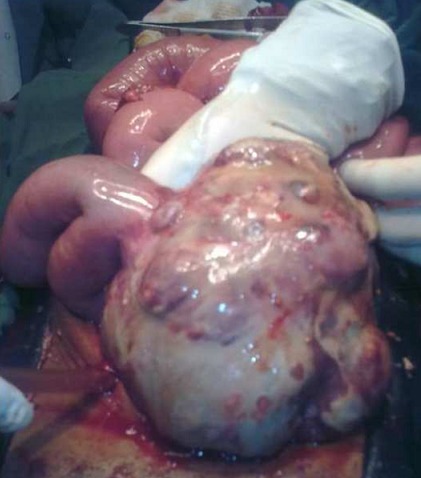
Aspect macroscopique de la tumeur iléale

**Figure 2:**
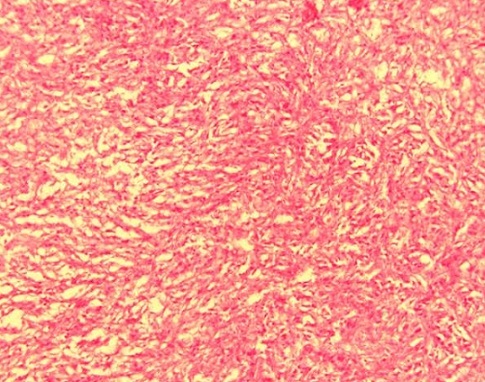
Prolifération de cellules fusiformes correspondant à la tumeur iléale (Hématéine-Eosine X10)

**Figure 3:**
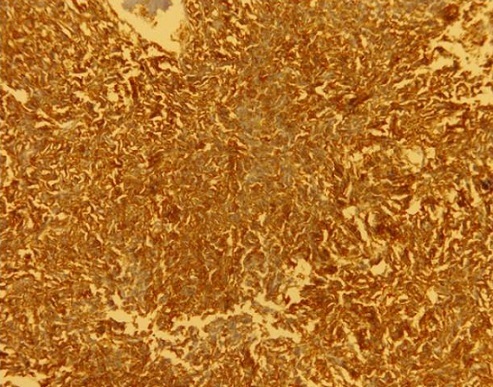
Immunomarquage positif au CD117 (c-kit) des cellules tumorales mésentériques (grossissement X10)

**Figure 4:**
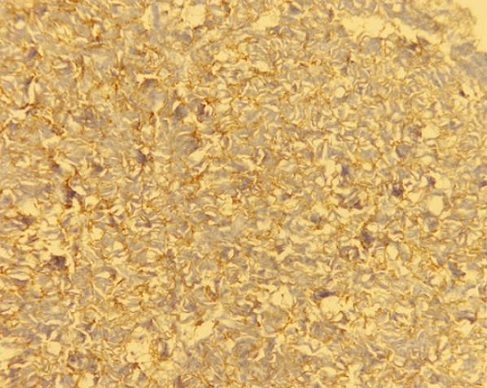
Immunomarquage positif au DOG1 des cellules tumorales mésentériques (grossissement X10)

Un homme âgé de 29 ans, sans antécédent particulier, était hospitalisé dans un service d'urologie au mois de mars 2010 pour une masse pelvienne douloureuse et pollakiurie évoluant depuis cinq mois. Après trois jours d'hospitalisation, le patient était opéré en urgence pour occlusion intestinale basse. A la laparotomie, une tumeur iléale était mise en évidence (Figure 1). Le foie était macroscopiquement sain et il n'y avait pas d'ascite ni de carcinose péritonéale. Une tumorectomie complète, sans curage ganglionnaire, suivie d'une anastomose iléo-iléale termino-terminale était réalisée. La tumeur, non rompue en peropératoire, était de couleur blanc grisâtre, de consistance molle à ferme. Elle était bien circonscrite par une pseudo-capsule fibro-adipeuse. A la coupe, elle était charnue, focalement kystisée, remaniée par la nécrose et l'hémorragie. L'examen histologique évoquait le diagnostic d'une GIST primitive caractérisée par une prolifération de cellules fusiformes organisées en faisceaux s'entrecroisant anarchiquement (Figure 2). Les marges de résection passaient en tissus sains. L'examen immunohistochimique faisait défaut. Les suites opératoires étaient simples. Après exeat, le patient était perdu de vue et ne pouvait pas bénéficier d'un complément thérapeutique par le Glivec^®^ (Imatinib). En février 2011, il était admis en chirurgie viscérale pour ballonnement et masse pelviens d’évolution rapidement progressive. A l'examen clinique, son état général était assez bon. L'inspection mettait en évidence une voussure hypogastrique et la palpation une défense abdominale diffuse. La biologie standard était normale. L’échographie abdominale révélait une masse extra-vésicale peu homogène, à contours réguliers et à limites moins nettes. L'origine et le siège de cette masse ne pouvaient pas être déterminés avec certitude. L'hypothèse d'une récidive locorégionale en rapport avec la tumeur iléale réséquée était évoquée. La réalisation d'une TDM était impossible par manque de moyens financiers. Le patient était opéré en urgence différée. L'exploration par laparotomie révélait une tumeur mésentérique assez volumineuse. Il n'y avait pas d'ascite. L'intestin grêle, le côlon, le foie ainsi que les épiploons étaient indemnes de lésions. Une tumorectomie en monobloc était décidée mais la résection était incomplète à cause d'une hémorragie importante induite par l'exérèse in situ. La masse tumorale avait le même profil histologique que la tumeur iléale. L'index mitotique était de 15 mitoses par 50 champs au fort grossissement (CFG). L’étude immunohistochimique mettait en évidence un immunomarquage fortement positif des cellules tumorales pour la vimentine, le c-kit (Figure 3) et le DOG1 (Figure 4). L'indice de prolifération (Ki-67 ou Mib-1) était évalué à 18%. Le diagnostic retenu était une tumeur stromale mésentérique à fort potentiel agressif, correspondant à une récidive métastatique de la tumeur iléale. Les suites opératoires étaient simples et le patient était adressé en oncologie pour complément thérapeutique par le Glivec^®^ (400mg/j). Le recul à 12 mois retrouvait un patient asymptomatique; aucune récidive locale ni de métastase n'a été observée.

## Discussion

Les GIST iléales sont relativement fréquentes [2, 3] et de mauvais pronostic surtout dans les formes tardives [3]. Actuellement, leur prise en charge est bien codifiée sauf dans les centres mal équipés comme le notre en raison des signes cliniques qui sont moins spécifiques et des moyens d'investigation performants qui font défaut. Sur le plan clinique, ces tumeurs peuvent être asymptomatiques, de découverte fortuite au décours d'un examen morphologique ou d'une chirurgie [1, 2, 4]. Certains symptômes sont toutefois révélateurs mais inconstants: hémorragie digestive, masse pelvienne, douleurs abdominales. La survenue d'une occlusion intestinale avec compression vésicale peut être révélatrice comme cela avait été le cas dans notre observation. Cette compression locorégionale est surtout expliquée par le glissement intermittent de l'intestin grêle dans le pelvis sous l'effet de la pesanteur et de la masse tumorale qui entre en contact avec le dôme vésical. Cette proximité anatomique explique la survenue d'une atteinte vésicale déjà observée chez certains patients [2].

Sur le plan paraclinique, l'exploration endoscopique utilisant la vidéocapsule est de pratique courante dans la prise en charge des tumeurs iléales à croissance intra-luminale [4]. Cette méthode n'a pas été réalisée chez notre patient par manque de matériels et de moyens financiers. L'autre option était la TDM mais notre patient ne pouvait pas en bénéficier. Elle est surtout indispensable en préopératoire pour caractériser le développement exophytique de la masse tumorale, d'apprécier le caractère résécable ou non des lésions, de détecter une éventuelle métastase ou de carcinose péritonéale et de choisir la voie d'abord chirurgicale [2]. Dans notre cas, le diagnostic de certitude ne pouvait être évoqué qu'en peropératoire en visualisant la masse tumorale à croissance externe. Sur le plan thérapeutique, le traitement de référence des GIST localisées est l'exérèse chirurgicale complète avec des marges saines [2, 3] éventuellement associée à un complément thérapeutique ciblé selon le degré de leur agressivité. La résection est souvent effectuée par laparotomie ou par voie laparoscopique [2–4], la résection endoscopique étant une option exceptionnelle [2]. L'avantage avec la chirurgie mini-invasive, non proposée chez notre patient par manque d'expérience et de matériels, serait de réduire à la fois la durée opératoire et le séjour hospitalier avec une cicatrice abdominale esthétique. En oncologie médicale, l'Imatinib (Glivec^®^) est souvent indiqué dans les GIST localisées et à fort potentiel malin ou avancées qu'elles soient non résecables, métastatiques ou récidivantes [1]. Dans notre cas, l'absence d'un tel traitement pourrait être à l'origine d'une rechute métastatique développée dans le mésentère. Généralement, la survenue de cette récidive correspond à un délai médian de 24 à 42 mois après la résection initiale [2, 5]. Elle est principalement corrélée à la taille tumorale, à son index mitotique, au stade localement avancé de la tumeur et à la survenue d'une effraction tumorale en peropératoire [2]. Cependant, il a été rapporté que la taille tumorale étant le seul facteur de risque statistiquement significatif [2]. En cas de GIST à potentiel malin, il existe un risqué élevé de récidive (10-50% selon les séries) même après une exérèse macroscopiquement complète [2, 5]. Mais, ce risque n'a aucun lien ni avec la réalisation ou non du curage ganglionnaire ni avec la réalisation d'une courte marge de résection. Car les métastases par cette voie sont rares [6] et même en intention curative, il n'existe pas de consensus sur les marges optimales de résection. Une marge de resection de 2 cm est toutefois raisonable. Le traitement adjuvant par l'Imatinib, lorsqu'il faisait défaut, semble constituer un autre facteur de risque métastatique [6]. Les sites métastatiques de prédilection sont représentés par le foie et le péritoine (mésentère, omentum) [2]. En cas de GIST métastatique développée dans le mésentère, le diagnostic différentiel se pose essentiellement avec la forme primitive d'EGIST (tumeur stromale extradigestive) mésentérique telle qu'elle est actuellement bien définie [1]. Il s'agit d'une tumeur mésenchymateuse rare développée à partir du tissu mou abdominal; elle est considérée primitive quand elle ne présente aucune attache avec le tube digestif [7, 8]. Les EGIST primitives ou métastatiques surexpriment le CD117 et présentent des profils de mutation des gènes c-kit et PDGFRA semblables aux GIST [8]. Actuellement, la protéine DOG1, positive chez notre patient, est proposée comme un marqueur sensible et spécifique des GIST [9]. Ce marqueur possède un intérêt particulier pour le diagnostic des GIST associées à une mutation du gène PDGFRA, où la protéine KIT est indétectable dans plus de 60% des cas, alors que DOG1 y semble constamment exprimée [1].

Par ailleurs, à l'opposé des GIST, l'histogénèse des EGIST primitives est encore mal élucidée. L'expression du c-kit par ces tumeurs suggère la présence de cellules interstitielles de Cajal en dehors du tractus digestif ou plutôt la capacité des cellules mésenchymateuses d'exprimer ce même phénotype d'une façon aberrante [7, 8]. Miettinen et al. pensent que les EGIST mésentériques primitives dériveraient de l'intestin puis s'en détachent au cours de leur développement [10]. Leur symptomatologie clinique est souvent tardive [10] du fait de leur siège qui est assez profond. Les circonstances de découvertes sont la perception d'une masse ou de douleurs abdominales [6, 7] comme cela avait été le cas dans notre observation. L'apport de l'imagerie (TDM, IRM) est incontestable dans le diagnostic préopératoire [11] mais il faisait défaut chez notre patient. L'avantage avec la TDM ou l'IRM est de permettre une bonne visualisation de la tumeur [6] et de guider la ponction biopsique à l'aiguille fine à visée diagnostique. Selon Ortiz-Rey et al, ce geste relativement simple et bénéfique est d'indication courante dans la prise en charge des tumeurs stromales extradigestives [12]. L'impossibilité de pratiquer cette méthode était à l'origine de notre attitude classique basée sur la laparotomie exploratrice. Elle nous permettait de visualiser la tumeur, d’établir un bilan lésionnel complet et d’évaluer les possibilités d'exérèse à visée diagnostique, thérapeutique et pronostique. Compte tenu des définitions admises, la stratégie dans la prise en charge des GIST métastatiques ou primitives du mésentère respecte les indications opératoires d'exérèse et de thérapeutique ciblée [1].

En intention curative, l'exérèse chirurgicale complète avec des marges saines est le traitement de première intention des tumeurs stromales extradigestives non métastatiques [13]. Le traitement adjuvant par l'imatinib est tout à fait licite et indiqué dans les formes avancées ou métastatiques [1, 14]. Dans notre cas, le schéma thérapeutique était combiné car il n'y avait pas de métastase hépatique et en raison de la nature incomplète de l'exérèse tumorale. Comme dans les GIST, l’évaluation pronostique des EGIST est encore difficile à établir. D'une part, le système de grading histopronostique utilisé pour les GIST, combinant index mitotique et taille tumorale n'est pas extrapolable pour les EGIST, ces dernières étant le plus souvent de grande taille au moment du diagnostic [7, 8]. D'autre part, le siège de la tumeur semble constituer un facteur pronostique indépendant [7]; les EGIST mésentérique sont moins agressives que les autres localisations péritonéales. Yamamoto et al. [13] définissent trois grades pronostiques sur la base de l'index mitotique et de l'indice de prolifération tumorale Ki-67; un index mitotique inférieur à 5/50 CFG et/ou un indice de prolifération inférieur à 10% permettent de classer la tumeur en EGIST à faible risque de malignité. En revanche, une tumeur ayant un index mitotique supérieur ou égal à 5/50 CFG et/ou un indice de prolifération supérieur ou égal à 10% est considérée à haut risque de malignité. Dans notre cas, étant donné l'existence de ce risque et l'exérèse incomplète de la tumeur, il était prudent et bénéfique d'avoir adopté un traitement adjuvant par l'Imatinib qui nous a donné un résultat satisfaisant au bout de 12 mois. Cependant, son efficacité à long terme reste à évaluer [8]. En effet, depuis 2007, des tumeurs stromales extradigestives ont été incluses dans les essais randomisés de la phase III ayant évalué l'Imatinib chez les patients porteurs de GIST [14]. En 2011, un cas de GIST métastatique résistant à l'Imatinib a été rapporté. A travers cette observation, les auteurs ont démontré l'intérêt clinique à long terme du Sunitinib utilisé à titre adjuvant à la place de l'Imatinib [15].

## Conclusion

Les tumeurs stromales extradigestives métastatiques ou primitives sont des tumeurs mésenchymateuses rares ayant une similitude morphologique et phénotypique avec les GIST. La survenue d'une récidive métastatique dans sa forme mésentérique était observée en absence de traitement adjuvant ciblé chez un patient ayant développé une GIST iléale à fort potentiel malin. La TDM peut être proposée comme l'examen de référence pour détecter précocement une éventuelle récidive, permettant ainsi d’évaluer les possibilités d'exérèse qui est le seul traitement à visée curative. L'imatinib était un complément thérapeutique adapté et bénéfique mais son efficacité à long terme reste à évaluer. Le Sunitinib pourrait être une alternative thérapeutique prometteuse.
